# Long-term camera trap monitoring of mammalian diversity and activity rhythms of threatened species in Xianxialing Nature Reserve, Zhejiang Province, China

**DOI:** 10.3897/BDJ.14.e208779

**Published:** 2026-09-08

**Authors:** Zhu-Cheng Yu, Xiao-Yi Wang, Chang Gao, Ke Wang, Mei-Fang Wang, Bai-Wei Gao, Wei Zhao, Si-Han Wu, Zhe-Ping Xu, Xiao Zhou, Lin-Li Xu, Jie Yu, Lotanna Micah Nneji, Jin-Min Chen, Wei Jin, Ping Shin Lee

**Affiliations:** 1 Jiangshan Xianxialing Nature Reserve Management Center of Zhejiang, Jiangshan 324100, China Jiangshan Xianxialing Nature Reserve Management Center of Zhejiang Jiangshan 324100 China; 2 The Anhui Provincial Key Laboratory of Biodiversity Conservation and Ecological Security in the Yangtze River Basin, College of Life Sciences, Anhui Normal University, Wuhu 241000, Anhui, China The Anhui Provincial Key Laboratory of Biodiversity Conservation and Ecological Security in the Yangtze River Basin, College of Life Sciences, Anhui Normal University Wuhu 241000, Anhui China; 3 Zhejiang Forest Resource Monitoring Center, Hangzhou 310020, China Zhejiang Forest Resource Monitoring Center Hangzhou 310020 China; 4 Department of Information Resources Management, School of Economics and Management, University of Chinese Academy of Sciences, Beijing 100190, China Department of Information Resources Management, School of Economics and Management, University of Chinese Academy of Sciences Beijing 100190 China; 5 National Science Library, Chinese Academy of Sciences, Beijing 100190, China National Science Library, Chinese Academy of Sciences Beijing 100190 China; 6 Zhejiang Forestry Survey Planning and Design Company Limited, Hangzhou 310020, China Zhejiang Forestry Survey Planning and Design Company Limited Hangzhou 310020 China; 7 Department of Biology, Howard University, Washington DC 20059, DC, United States of America Department of Biology, Howard University Washington DC 20059, DC United States of America

**Keywords:** community composition, conservation, diel rhythm, infrared camera, mammals

## Abstract

**Background:**

Research on mammalian diversity and activity rhythms is critical for understanding biological adaptation, evolutionary processes and the development of effective ecological conservation strategies. Xianxialing Nature Reserve in Zhejiang Province is an area with rich mammalian biodiversity, yet no study has been conducted on mammalian activity rhythms in the Reserve. This study aims to systematically monitor and document mammalian diversity and characterise the activity rhythms of threatened species in the Xianxialing Nature Reserve using infrared camera traps.

**New information:**

Between January 2021 and November 2023, this survey used 97 continuously monitored infrared cameras, generating totals of 71,879 effective camera-days of data. In total, 24,919 effective records were obtained, representing 12 independently detected non-rodent mammal species belonging to seven families, 10 genera and four orders. Based on the Relative Abundance Index (RAI), the five most abundant species were Reeves' Muntjac (*Muntiacus reevesi*), Masked Palm Civet (*Paguma larvata*), Hog Badger (*Arctonyx collaris*), Chinese Ferret-badger (*Melogale moschata*) and Rhesus Macaque (*Macaca mulatta*). Compared with diurnal species, nocturnal species, such as the Masked Palm Civet, showed a preference for lower-altitude habitats during winter. These spatial distribution patterns can be attributed to a combination of thermoregulatory strategies and other ecological drivers. Overall, the findings provide an objective assessment of the current status of mammalian diversity in Xianxialing Nature Reserve. Studying mammal activity rhythms offers baseline data and practical insights for the dynamic conservation of nationally protected species in China's subtropical nature reserves.

## Introduction

Mammals are the important indicator groups for biodiversity conservation and assessment due to their wide distribution ranges and good adaptability to diverse habitat types (Xiao et al. 2017, Li et al. 2018). However, in recent years, mammals have become increasingly vulnerable to external stressors associated with anthropogenic disturbances, such as habitat fragmentation (Liu et al. 2018, Wang et al. 2022, Duan et al. 2025). According to the International Union for Conservation of Nature (IUCN) Red List of Threatened Species, approximately 26% of all mammalian taxa are currently classified as threatened (IUCN 2026). Thus, there is a need for systematic monitoring of mammalian diversity, population status and behavioural ecology to support effective conservation planning. In mammalian diversity assessments and community surveys of mammals, the activity rhythm is a critical parameter for evaluating the population status of wild animals, because it provides insights into the ecological habits and behavioural patterns (Amstrup and Beecham 1976, Li et al. 2022, Li et al. 2023). Studies on activity rhythm mainly investigate animal activity intensity and variation across different time periods and seasons, reflecting important ecological processes, such as individual adaptation, circadian behaviour and seasonal movement patterns (Azevedo et al. 2018).

Infrared cameras are non-invasive, highly concealed and sustainable tools that are minimally affected by environmental conditions or human interference, making them particularly useful for wildlife research and biodiversity surveys (Wu et al. 2012). They are now widely used in animal ecology to investigate wildlife activity rhythms, including diel activity patterns, population size, species distribution and community structure (Fu et al. 2020, Zhang 2022, Fisher 2023). For example, previous studies used camera traps to survey mammal activity rhythms: Alemu et al. in Ethiopia, Gui et al. in Hunan and Yang et al. in Chongqing, targeting different taxa and temporal scales (Yang et al. 2021, Alemu et al. 2024, Gui et al. 2025).

Owing to their favourable physical and geographic conditions, the Xianxialing Nature Reserve supports rich mammalian diversity and is particularly notable for its rare and endangered species, making the area one of the most important biodiversity-rich regions in Zhejiang Province, with high conservation value and great scientific research significance (Yu et al. 2023). Although progress has been made in some regions in recent years (Nie et al. 2025), existing studies in Xianxialing Nature Reserve mainly focus on species diversity surveys (Chen et al. 2023, Yu et al. 2023). To date, no systematic and comprehensive study has examined mammalian activity rhythms within this Reserve.

## General description

### Purpose

This study uses infrared camera traps to document mammal community composition and characterise the activity rhythms of nine threatened species in Xianxialing Nature Reserve, providing baseline data to provide information for conservation strategies.

## Project description

### Study area description

Xianxialing Nature Reserve is located between 28°15'47"N to 28°27'40"N latitude and 118°20'14"E to 118°40'50"E longitude. The region has an average temperature of 15.0℃, annual precipitation of 1650–2200 mm and average altitude of 1000 m. Owing to its complex topography, this Reserve experiences four distinct seasons and pronounced microclimatic variation (Chen et al. 2020).

### Design description

**Figure 1.**
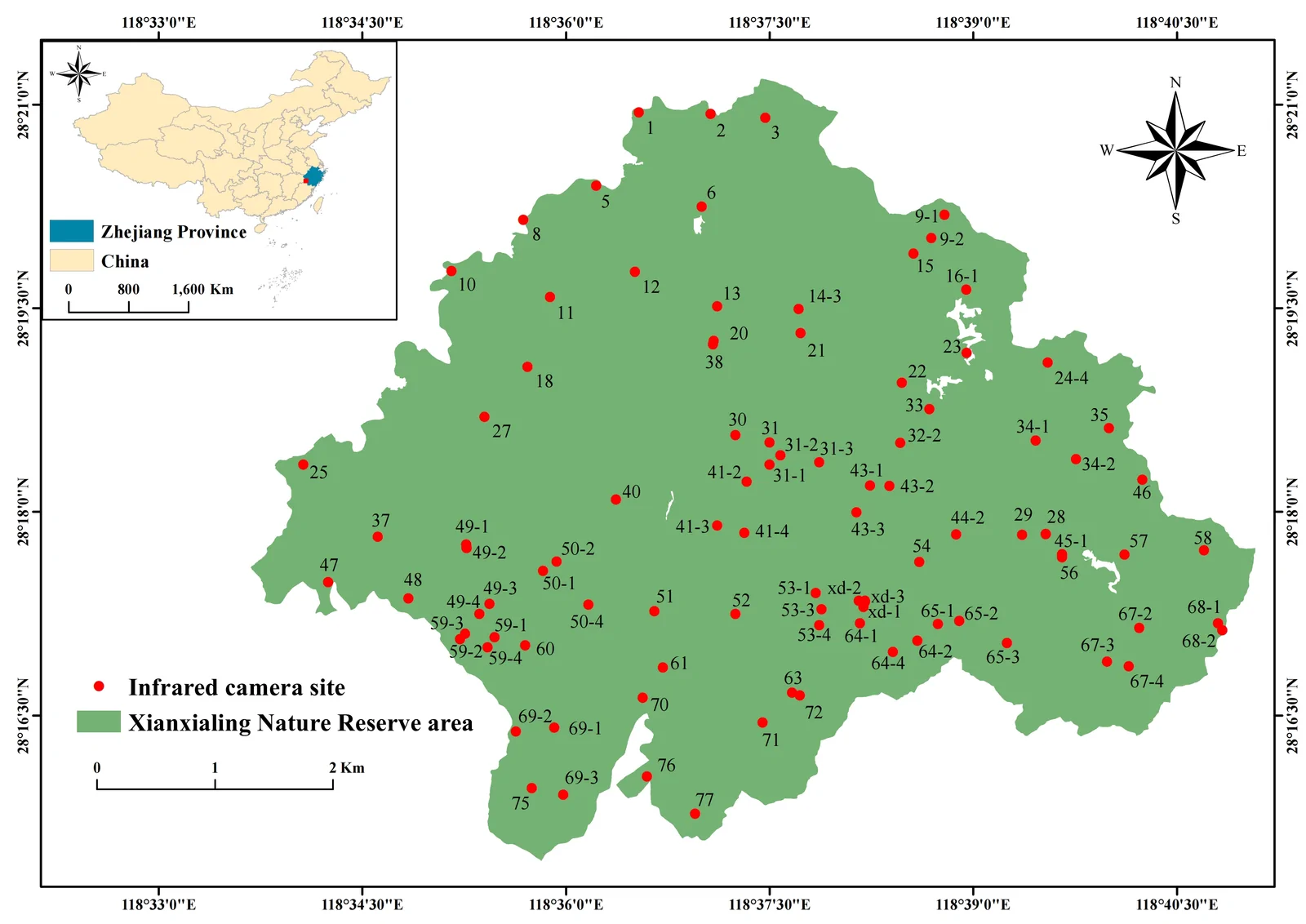
Locations of camera traps in the Xianxialing Nature Reserve.

From January 2021 to November 2023, we deployed 97 infrared-triggered cameras for wildlife monitoring (Fig. 1). Camera stations were preferentially established in open understorey near water sources and selected, based on visible animal signs (e.g. trails, faeces, nests, foraging marks, scratches and feathers). Associated metadata–coordinates, ID, timestamp, elevation and habitat type, were recorded concurrently.

### Funding

This research was funded by the National Natural Science Foundation of China (NSFC 32001222; 32470458; 32670594) and the Innovation and Entrepreneurship Training Programme for Undergraduate Students (2025026111; 2025026611; 2026000811).

## Sampling methods

### Sampling description

The study area was divided into a 1 km × 1 km grid. Species were identified from infrared camera-trap data. Cameras were mounted on tree trunks 80–100 cm above the ground, positioned approximately 45° to animal trails and spaced at least 100 m apart. Camera lenses were kept parallel to the ground or tilted downwards by no more than 5°. Vegetation and other obstructions were cleared before deployment. All cameras operated continuously for 24 hours per day and were set to capture three photographs and one video per trigger.

### Quality control

To ensure accurate species identification, all infrared camera photos and videos were systematically reviewed and compared with voucher specimens and published literature (Liu et al. 2022). Species names, including Chinese common names and Latin names, were verified using the Checklist of Mammals of China (Wei et al. 2025). Conservation statuses were assigned using the IUCN Red List of Threatened Species (IUCN 2026). Records that could only be identified as family (Muridae or Sciuridae) and could not be resolved to species were excluded from the final analysis.

### Step description

Infrared camera data (photos and videos) were archived by camera ID, with each camera assigned a dedicated folder. Metadata (capture date, time and file name) were extracted to Excel using Picture Information Extractor. After metadata extraction, species identification was conducted separately for each camera and only valid photographs were retained for analyses. To ensure data independence, consecutive records of the same species captured by the same camera within a 30-minute interval were counted as a single independent valid monitoring event (Liu et al. 2018). One effective camera working day was defined as a 24-hour deployment of a single camera station (He et al. 2016).

The Relative Abundance Index (RAI) for each species was calculated as the proportion of its independent valid photographs relative to the total number of independent valid photographs recorded for all species (Wu et al. 2012, Chen et al. 2019). To assess the differences in species diversity between low- and high-altitude zones, we calculated Hill numbers with q = 0 (species richness), q = 1 (exponential of Shannon entropy) and q = 2 (inverse Simpson index) (Shannon 1948, Simpson 1949, Ricotta and Feoli 2024). Rarefaction curves were generated using EstimateS 9.1.0 and sampling adequacy was evaluated using the Chao 1 method (Colwell 2019). To quantify diel activity patterns, the night-time relative abundance index (NRAI) was calculated. Species were classified as nocturnal when their NRAI exceeded 13/24 (Han et al. 2022). Kernel density analysis was performed using the "activity" and "overlap" packages in RStudio to examine diurnal and seasonal activity rhythms of each species (Gao et al. 2024).

The altitude of the survey area ranged from 388 m to 1460 m. Due to the limited altitude range of camera deployment, the study area was divided into low- (388–943 m) and high-altitude (944–1460 m) zones, based on altitude gradients and vegetation types (Huang 2023). Seasons were defined as spring (March-May), summer (June-August), autumn (September-November) and winter (December-February). One-way ANOVA was performed using SPSS, with the significance threshold set at P = 0.05, to test for seasonal differences in elevation preference amongst species (Lu et al. 2003).

## Geographic coverage

### Description

The Xianxialing Nature Reserve, Jiangshan, Zhejiang, China.

### Coordinates

28.26293 and 28.46115 Latitude; 118.33722 and 118.68063 Longitude.

## Taxonomic coverage

### Description

This study covered the following taxonomic groups: Class: Mammalia; Orders: Lagomorpha, Carnivora, Primates and Artiodactyla; Families: Leporidae, Suidae, Cervidae, Cercopithecidae, Mustelidae, Viverridae and Bovidae.

### Taxa included

**Table:** 

Rank	Scientific Name	Common Name
class	Mammalia	Mammals
order	Lagomorpha	Hares
order	Carnivora	Carnivores
order	Primates	Primates
order	Artiodactyla	Ruminants
family	Leporidae	Hares
family	Suidae	Pigs
family	Cervidae	Deers
family	Cercopithecidae	Monkeys
family	Mustelidae	Mustelids
family	Viverridae	Civets
family	Bovidae	Bovids

## Temporal coverage

**Data range:** 2021-1-01 – 2023-11-14.

## Usage licence

### Usage licence

Other

### IP rights notes

Creative Commons Attribution 4.0 International (CC-BY-4.0).

## Data resources

### Data package title

Camera trap survey of mammal diversity and activity rhythms of threatened species in Xianxialing Nature Reserve, Zhejiang Province, China

### Resource link

https://doi.org/10.15468/w9szht

### Alternative identifiers

https://www.gbifchina.org.cn/resource?r=wxy2000724

### Number of data sets

1

### Data set 1.

#### Data set name

Camera trap survey of mammal diversity and activity rhythms of threatened species in Xianxialing Nature Reserve, Zhejiang Province, China

#### Data format

Darwin Core Archive

#### Download URL

https://www.gbifchina.org.cn/archive.do?r=wxy2000724

#### Data format version

version 1.2

#### Description

This dataset has been published in GBIF (Wang and Chen 2026). All records with valid species identification are compiled in the table below. The dataset submitted to GBIF adopts the sample event dataset structure, comprising two tables: "Event" and "Occurrence". These data are published in Darwin Core Archive (DwC-A) format, a standard biodiversity data-sharing format typically consisting of one or more data tables. The core table contains 97 records (event IDs) and the extension table includes 24,919 occurrence records.

**Data set 1.** 

Column label	Column description
eventID (Event Core, Occurrence Extension)	A unique identifier linking events and occurrences.
samplingProtocol (Event Core)	The sampling method used.
samplingEffort (Event Core)	The number of camera-trap days expended during an event.
eventDate (Event Core, Occurrence Extension)	Date or date range the record was collected.
country (Event Core)	Country in which camera location occurs.
countryCode (Event Core, Occurrence Extension)	ISO code of the country.
decimalLatitude (Event Core, Occurrence Extension)	The geographic latitude, in decimal degrees.
decimalLongitude (Event Core, Occurrence Extension)	The geographic longitude, in decimal degrees.
geodeticDatum (Event Core, Occurrence Extension)	The reference point for the various coordinate systems used in mapping the Earth.
occurrenceID (Occurrence Extension)	Unique identifier of the record.
basisOfRecord (Occurrence Extension)	The specific nature of the data record.
occurrenceStatus (Occurrence Extension)	A statement about the presence or absence of a taxon at a location.
kingdom (Occurrence Extension)	Kingdom name in which the taxon is classified.
phylum (Occurrence Extension)	Phylum name in which the taxon is classified.
class (Occurrence Extension)	Class name in which the taxon is classified.
order (Occurrence Extension)	Order name in which the taxon is classified.
family (Occurrence Extension)	Family name in which the taxon is classified.
scientificName (Occurrence Extension)	The full scientific name includes author and year.
taxonRank (Occurrence Extension)	The taxonomic rank of the most specific name in the scientificName.
type (Occurrence Extension)	The nature or genre of the resource.
eventRemarks (Occurrence Extension)	The date of the image capture.
coordinateUncertaintyInMetres (Event Core)	Uncertainty of the coordinates, in metres.

## Additional information

### Result

**Figure 2.**
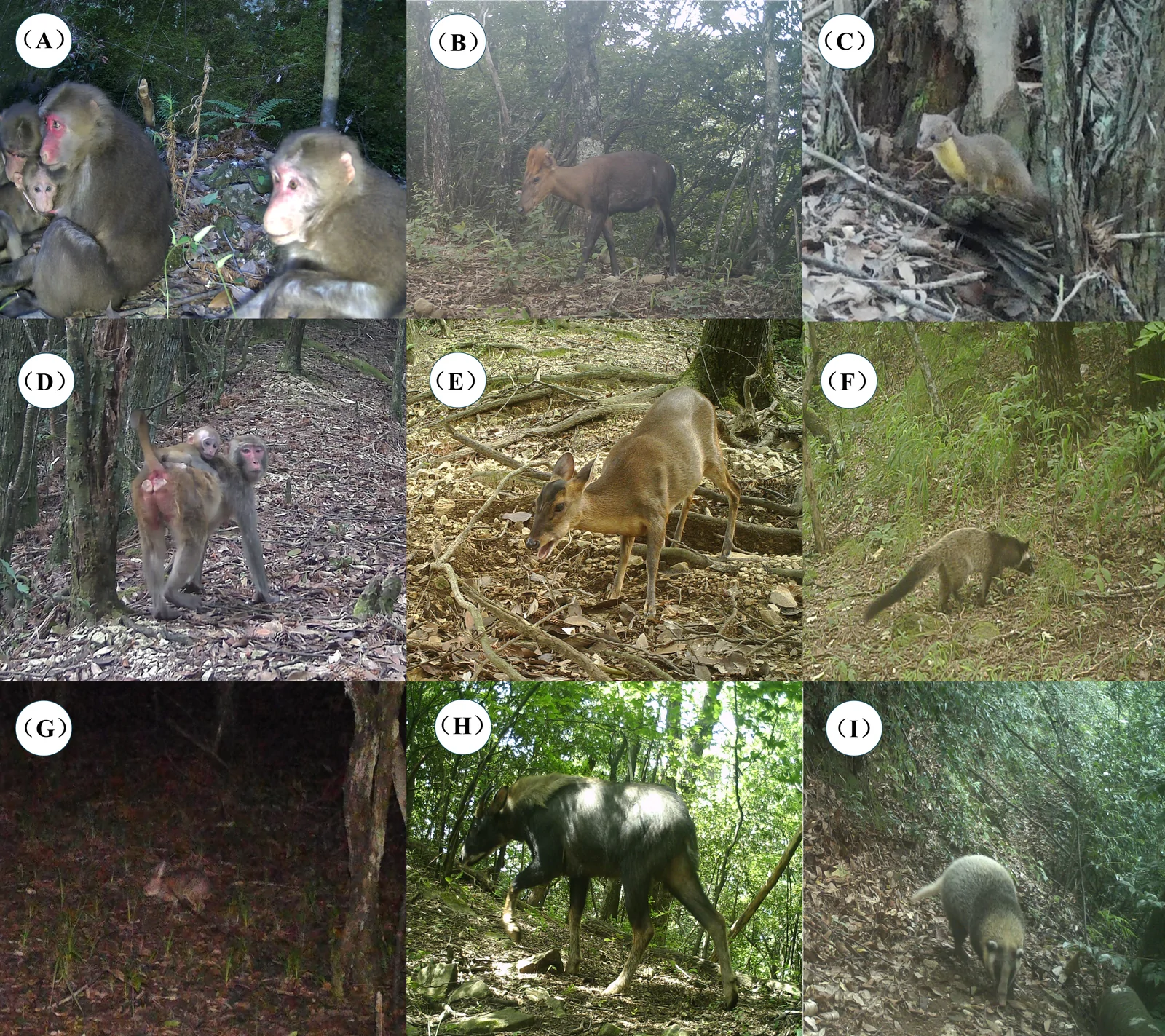
Representative mammalian species captured by infrared cameras at the Xianxialing Nature Reserve: **A** Tibetan Macaque (*Macaca thibetana*, summer, 05:13:57); **B** Black Muntjac (*Muntiacus crinifrons*, summer, 16:50:32); **C** Yellow-bellied Weasel (*Mustela kathiah*, autumn, 07:00:01); **D** Rhesus Macaque (*Macaca mulatta*, summer, 14:33:27); **E** Reeves' Muntjac (*Muntiacus reevesi*, summer, 10:42:20); **F** Masked Palm Civet (*Paguma larvata*, summer, 11:08:06); **G** Chinese Hare (*Lepus sinensis*, summer, 20:13:29); **H** Chinese Serow (*Capricornis sumatraensis*, summer, 14:19:58); **I** Hog Badger (*Arctonyx collaris*, summer, 15:50:19).

A total of 97 infrared cameras were deployed for continuous monitoring, generating 71,879 effective camera-days and capturing 24,919 independent valid photographs of wild mammals (Fig. 2). Excluding records identified only as Muridae and Sciuridae, 12 mammal species were identified, belonging to four orders, seven families and 10 genera. These represent 19.05% of all mammal species recorded in the Xianxialing Reserve, based on the species checklist from The Comprehensive Scientific Expedition Report (Suppl. material 2). The identified species included five Carnivora, four Artiodactyla, two Primates and one Lagomorpha. Amongst the recorded species, Black Muntjac (*Muntiacus crinifrons*) is Class I nationally protected species, while Chinese Serow (*Capricornis sumatraensis*), Rhesus Macaque (*M. mulatta*) and Tibetan Macaque (*Macaca thibetana*) are listed as Class II nationally protected species. According to the IUCN Red List, Black Muntjac, Chinese Serow and Hog Badger (*A. collaris*) are classified as Vulnerable (VU), whereas Tibetan Macaque is classified as Near Threatened (NT). Based on descending Relative Abundance Index (RAI) values, the recorded species were Reeves' Muntjac (*M. reevesi*, 20.765), Masked Palm Civet (*P. larvata*, 1.906), Hog Badger (*A. collaris*, 1.434), Chinese Ferret-badger (*M. moschata*, 0.985), Rhesus Macaque (*M. mulatta*, 0.584), Tibetan Macaque (*Macaca thibetana*, 0.328), Chinese Hare (*Lepus sinensis*, 0.269), Chinese Serow (*C. sumatraensis*, 0.214), Black Muntjac (*M. crinifrons*, 0.156), Wild Boar (*Sus scrofa*, 0.024), Yellow-bellied Weasel (*Mustela kathiah*, 0.001) and Asian Badger (*Meles leucurus*, 0.001) (Table 1).

**Figure 3.**
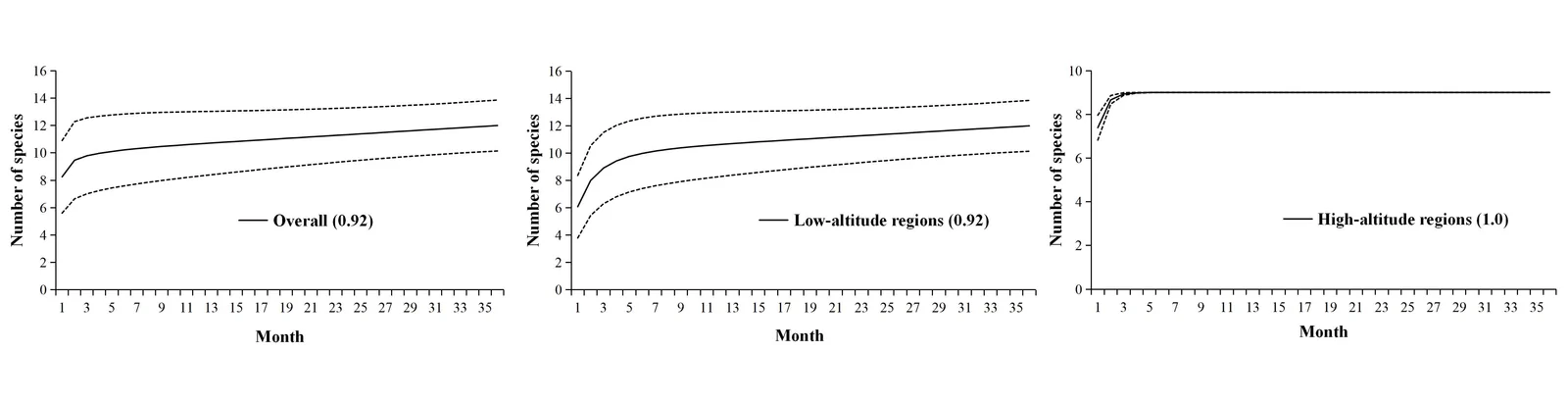
Expected species richness curves for overall, low-altitude and high-altitude areas in Xianxialing Nature Reserve. Dashed lines indicate the 95% confidence interval and Chao 1 estimates 0.92, 0.92 and 1.00, respectively.

Camera-based rarefaction analysis showed that species accumulation increased rapidly at first and then gradually levelled off, although it did not reach full saturation. The Chao 1 estimator indicated a sampling coverage of 0.92 for Xianxialing Reserve, suggesting that the survey captured most, but not all, mammalian species present in the area. When stratified by altitude, the low-altitude zone exhibited a species accumulation pattern consistent with the overall trend, whereas the high-altitude zone reached a stable asymptotic value (1.0) (Fig. 3).

The study sites were divided into low-altitude and high-altitude zones. Comparing zone-specific Hill numbers showed higher mammal diversity and evenness at low altitudes (Table 2).

**Figure 4.**
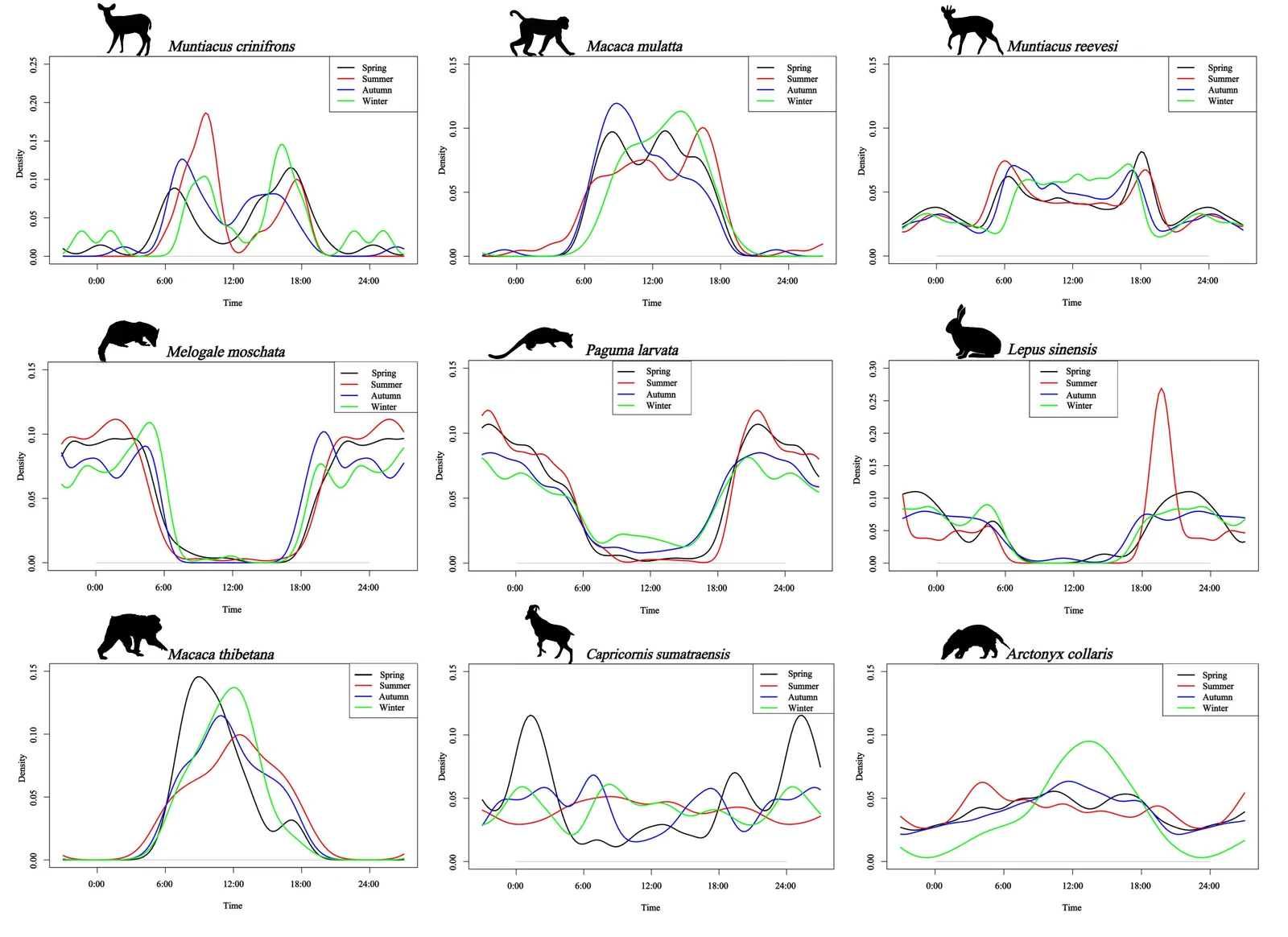
Daily activity patterns of nine threatened species in the Xianxialing Nature Reserve.

Diel activity rhythm analysis revealed four primary activity patterns amongst mammals in the study area (Fig. 4). Reeves' Muntjac, Black Muntjac and Rhesus Macaque exhibited a typical bimodal "M-shaped" activity pattern, although peak activity times varied seasonally. Reeves' Muntjac was primarily active at dawn and dusk in spring, summer and autumn, with an additional mid-day activity peak in summer; Black Muntjac showed seasonal variation in peak activity, with peaks at 06:00 h and 18:00 h in spring, at 18:00 and 06:00–12:00 h in summer, at 06:00 h in autumn and at 06:00–12:00 h and 12:00–18:00 h in winter. Rhesus Macaque peaked at 06:00 h and 12:00 h in spring, with the highest activity peaks at 18:00 h in summer, 06:00 h in autumn and 12:00 h in winter. Tibetan Macaque was mainly diurnal, with peak activity at 12:00 h in summer and winter and at 06:00–12:00 h in spring and autumn, together with a secondary peak at 18:00 h in spring. Chinese Hare, Masked Palm Civet and Chinese Ferret-badger followed a "U-shaped" activity pattern, with activity peaks before 06:00 h and after 18:00 h throughout the year. Chinese Serow and Hog Badger showed irregular activity patterns. Chinese Serow peaked only around midnight in spring, whereas Hog Badger peaked at 12:00 h in winter.

**Figure 5.**
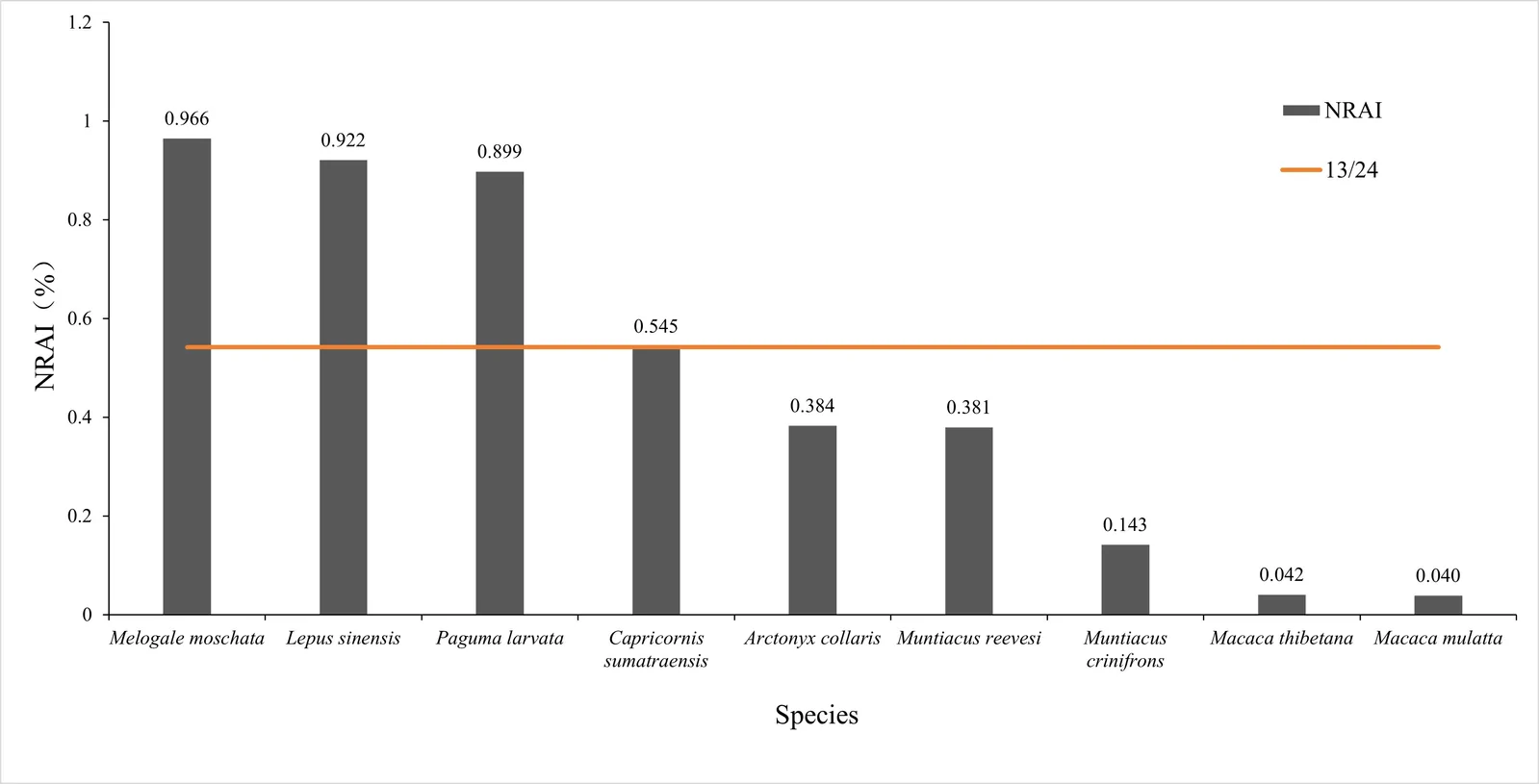
Nocturnal indices of nine threatened species in the Xianxialing Nature Reserve.

The nocturnal activity index (NRAI) values for the nine species (Chinese Ferret-badger, Chinese Hare, Masked Palm Civet, Chinese Serow, Hog Badger, Reeves' Muntjac, Black Muntjac, Tibetan Macaque and Rhesus Macaque) were as follows: 0.966, 0.922, 0.899, 0.545, 0.384, 0.381, 0.143, 0.042 and 0.040 (Fig. 5). Chinese Ferret-badger, Chinese Hare and Masked Palm Civet had NRAI values greater than 13/24, indicating nocturnal activity. Hog Badger, Reeves' Muntjac, Black Muntjac, Tibetan Macaque and Rhesus Macaque had NRAI values below 13/24, indicating diurnal activity. Chinese Serow had an NRAI of 0.545, close to the classification threshold, suggesting weak nocturnality.

**Figure 6.**
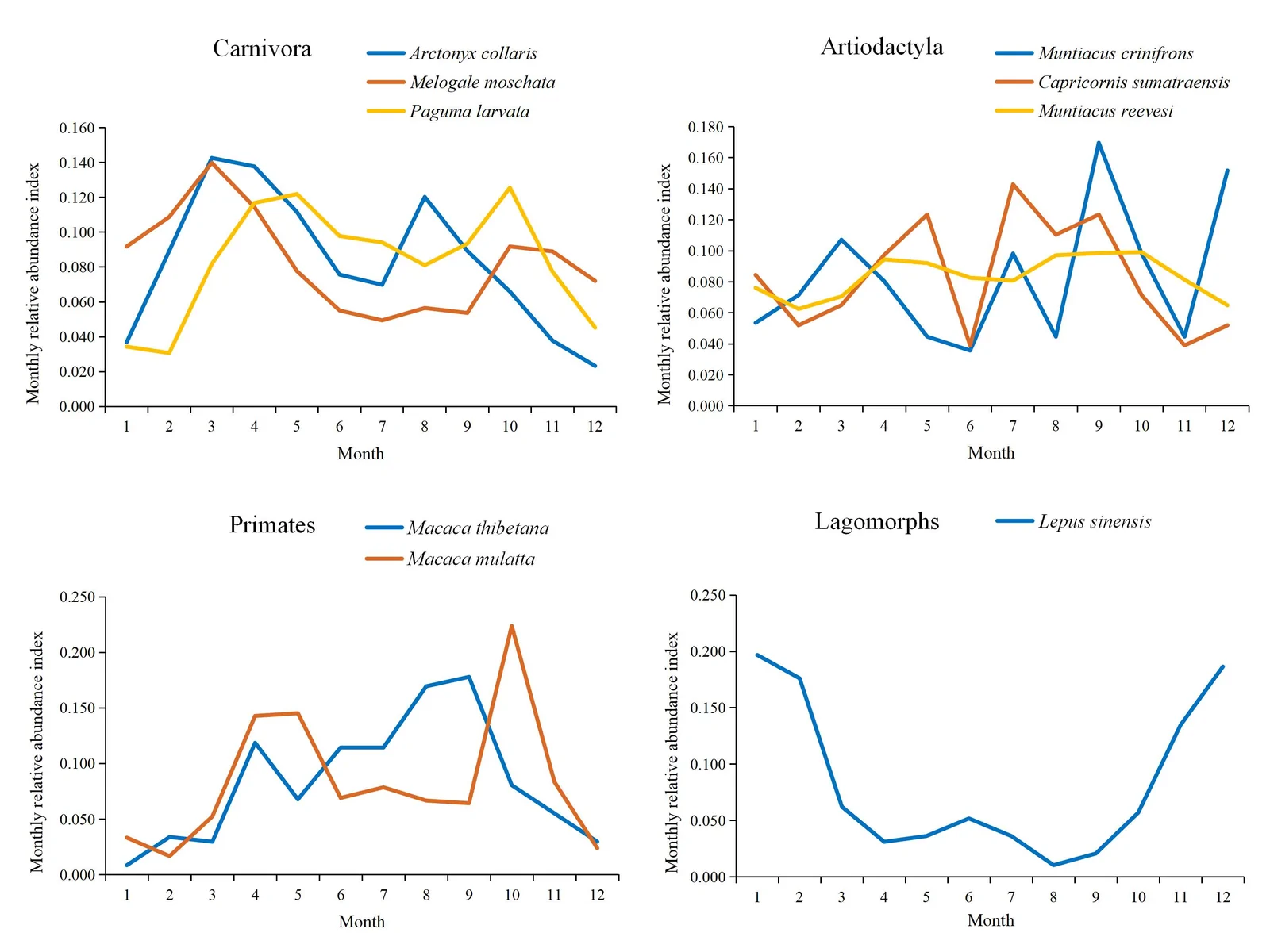
Annual activity patterns of nine threatened species in the Xianxialing Nature Reserve.

Monthly activity intensity was analysed at different taxonomic order levels (Fig. 6). Amongst Primates, Tibetan Macaque activity peaked in September and lowest in January, with higher activity in summer and autumn than in winter and spring. Rhesus Macaque peaked in October and reached the lowest level from January to February. Amongst Artiodactyla, Black Muntjac had a main activity peak in September, a secondary peak in December, lowest activity in August and a marked increase in autumn. Chinese Serow activity peaked in July and September and reached the lowest level in June. Reeves' Muntjac showed a relatively smooth activity curve with minor year-round fluctuations. Amongst Carnivora, Hog Badger and Chinese Ferret-badger peaked in March and then declined to their lowest levels in winter, showing a pattern of high spring activity and low winter activity. Masked Palm Civet showed a main activity peak from April to May, a secondary peak in October and a slight decline in summer, particularly from July to August, indicating a bimodal spring-autumn rhythm. Amongst Lagomorpha, Chinese Hare activity peaked in January and December, was lowest in August and was significantly higher during winter activity.

**Figure 7.**
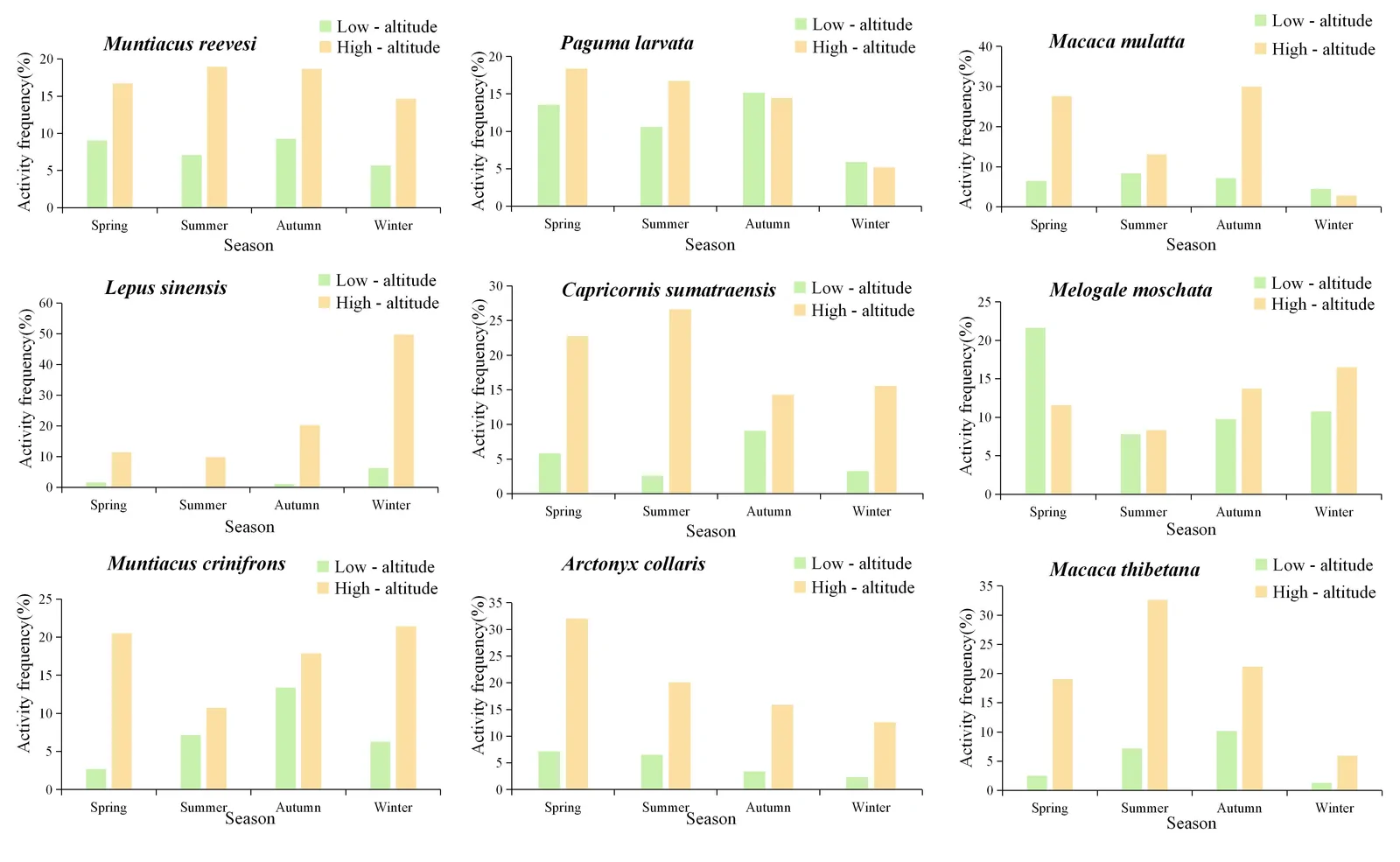
Seasonal altitudinal migration of nine threatened species in Xianxialing Nature Reserve.

Seasonal altitudinal preferences varied amongst species (Suppl. material 1). Tibetan Macaque, Reeves' Muntjac, Hog Badger, Black Muntjac, Chinese Hare and Chinese Serow preferred high-altitude habitats throughout the year. Masked Palm Civet preferred high altitudes in spring and summer, but shifted to low altitudes in autumn and winter. Chinese Ferret-badger preferred high altitudes in summer, autumn and winter, but used low altitudes in spring. Rhesus Macaque preferred high altitudes in spring, summer and autumn, but moved to lower altitudes in winter (Fig. 7).

### Discussion

Xianxialing Provincial Nature Reserve is highly biodiverse and harbours numerous rare species (Ji et al. 2022). This study provides the first camera-trap survey of threatened mammal activity rhythms since the Reserve's establishment, recording 12 mammal species, including nationally protected Tibetan Macaque, Rhesus Macaque, Black Muntjac and Chinese Serow (Chen et al. 2020). These findings highlight the high conservation and scientific value of the Reserve (Song et al. 2017). The rarefaction curve confirmed sufficient sampling effort and Reeves' Muntjac dominated small- to medium-sized ground-dwelling mammals, consistent with findings from other Chinese subtropical reserves (Fu et al. 2020, Hu et al. 2023). Overall, this dataset provides a valuable baseline for future ecological research, monitoring and conservation management in Xianxialing Nature Reserve, while also demonstrating the conservation importance of the region and the effectiveness of camera-trap surveys.

Activity rhythms showed clear interspecific and seasonal differences, reflecting adaptive strategies to local environments. For instance, Chinese Ferret-badger, Chinese Hare and Masked Palm Civet exhibit typical nocturnal activity patterns, consistent with their nocturnal foraging niches (Li et al. 2022, Huang 2023, Xiao et al. 2024). This pattern may represent a behavioural adaptation that helps these species avoid anthropogenic disturbance during daylight hours, such as the regular ranger patrols in the Xianxialing Nature Reserve (Hou et al. 2022). In contrast, Reeves' Muntjac and Black Muntjac showed a typical bimodal (crepuscular) daily activity pattern (Hu et al. 2022, Zhao et al. 2026). The preference for crepuscular periods may represent a form of temporal niche optimisation strategy, allowing species to use the suitable light and temperature conditions at dawn and dusk while avoiding potential daytime risks and improve foraging efficiency (Camp et al. 2018, Chai et al. 2024). Furthermore, the pronounced mid-day activity peak of Hog Badger during winter reflects adaptation to local conditions. Under limited winter food availability, Hog badger may increase daytime activity to meet energy demands (Li et al. 2023, Zhao et al. 2024).

Altitudinal migration is an important spatial adaptive strategy that animals use to cope with seasonal environmental changes (John and Post 2021, Huang 2023). Seasonal altitudinal migration reflects complex trade-offs amongst multiple ecological factors (Zhao et al. 2026). In this study, Masked Palm Civet exhibited clear vertical migration, preferring high-altitude habitats in spring and summer and shifting to low altitudes in autumn and winter. This "upward in summer, downward in winter" pattern may represent an adaptive response to seasonal temperature variation (Zeng et al. 2010, Yu et al. 2011). In contrast, Hog Badger, Black Muntjac, Chinese Hare and Chinese Serow occupied high-altitude habitats throughout the year (Zeng et al. 2009, Wang et al. 2022, Zhang et al. 2025), long-term adaptation to high-altitude environmental conditions, including lower temperatures and specific vegetation types (Si et al. 2023). These divergent altitudinal strategies may allow sympatric species to temporally partition resources across altitudinal zones, thereby reducing interspecific competition (Zhang 2022). Future studies should integrate high-resolution vegetation maps, quantified anthropogenic disturbance data and climate data to clarify the mechanisms underlying these ecological patterns.

## Supplementary Material

277C687A-4006-5389-81CA-F844AA2D82F4 10.3897/BDJ.14.e208779.suppl1 22702372 Supplementary material 1 Differences in the number of detections Data type Table Brief description Differences in the occurrence frequency of nine threatened species across different months and differences in elevation selection across different seasons File: oo_1713042.csv https://binary.pensoft.net/file/1713042 Xiao-Yi Wang

D3FB651B-209C-5682-9EF0-D91FB07C1AE5 10.3897/BDJ.14.e208779.suppl2 22702374 Supplementary material 2 List of mammal species Data type Table Brief description List of mammal species recorded at the Xianxialing Nature Reserve, Jiangshan of Zhejiang Province. File: oo_1695622.pdf https://binary.pensoft.net/file/1695622 Xiao-Yi Wang

## Figures and Tables

**Table 1.** Species diversity of mammals in the Xianxialing Nature Reserve.

Species	IUCN Red List	China Species Red List	National Key Protection Level	Endemic Species of China	Number of independent photos	Relative Abundance Index
**CARNIVORA**						
** Mustelidae **						
*Arctonyx collaris* F. Cuvier in É. Geoffroy Saint-Hilaire & F. Cuvier, 1825	VU	NT	-	-	1031	1.4344
*Melogale moschata* (J. E. Gray, 1831)	LC	NT	-	-	708	0.9850
*Meles leucurus* (B. H. Hodgson, 1847)	LC	NT	-	-	1	0.0014
*Mustela kathiah* B. H. Hodgson, 1835	LC	NT	-	-	1	0.0014
** Viverridae **						
*Paguma larvata* (C. E. H. Smith, 1827)	LC	NT	-	-	1370	1.9060
**ARTIODACTYLA**						
** Cervidae **						
*Muntiacus reevesi* (W. Ogilby, 1839)	LC	NT	-	√	20676	28.7650
*Muntiacus crinifrons* (P. L. Sclater, 1885)	VU	EN	Ⅰ	√	112	0.1558
** Bovidae **						
*Capricornis sumatraensis* (Bechstein, 1799)	VU	VU	Ⅱ	-	154	0.2142
** Suidae **						
*Sus scrofa* Linnaeus, 1758	LC	LC	-	-	17	0.0237
**PRIMATES**						
** Cercopithecidae **						
*Macaca thibetana* (A. Milne-Edwards, 1870)	NT	VU	Ⅱ	√	236	0.3283
*Macaca mulatta* (E. A. W. von Zimmermann, 1780)	LC	LC	Ⅱ	-	420	0.5843
**LAGOMORPHA**						
** Leporidae **						
*Lepus sinensis* J. E. Gray, 1832	LC	LC	-	-	193	0.2685

**Table 2.** Species diversity (Hill numbers) across altitude zones in Xianxialing Nature Reserve.

Altitude gradient	q = 0	q = 1	q = 2
Low altitude (388–943 m)	12	2.904	1.931
High altitude (947–1460 m)	9	2.282	1.477

## References

[B14232124] Alemu Nimona, Gadisa Tsegaye, Habtamu Tadesse (2024). Diversity, distribution, and relative abundance of Medium and Large-sized mammals in Chukala Mountain Forest, East Shoa Zone, Oromia, Ethiopia. BMC Zoology.

[B14232156] Amstrup Steven C., Beecham John (1976). Activity batterns of radio-collared black bears in Idaho. The Journal of Wildlife Management.

[B14237413] Azevedo F. C., Lemos F. G., Freitas‐Junior M. C., Rocha D. G., Azevedo F. C. C. (2018). Puma activity patterns and temporal overlap with prey in a Human‐modified landscape at Southeastern Brazil. Journal of Zoology.

[B14237423] Camp Meghan J., Shipley Lisa A., Milling Charlotte R., Rachlow Janet L., Forbey Jennifer S. (2018). Interacting effects of ambient temperature and food quality on the foraging ecology of small mammalian herbivores. Journal of Thermal Biology.

[B14238622] Chai Y, Mao X, Li G, Sun N (2024). Spatiotemporal niche relationship between Leopard and its sympatric Carnivores in the forest of Baizha, Qinghai Province. Acta Theriologica Sinica.

[B14237651] Chen L, Xiao W, Xiao Z (2019). Limitations of relative abundance indices calculated from camera-trapping data. Biodiversity Science.

[B14238647] Chen Z, Yu Z, Jin W (2020). The rare and endangered animals and plants in Jiangshan Xianxialing Nature Reserve.

[B14237660] Chen Z, Yu Z, Zhou X, Yu J, Wang M, Tong Z, Xu L (2023). Study on *Muntiacus
crinifrons* resources in Xianxialing Nature Reserve，Jiangshan. Anhui Agricultural Sciences.

[B14264100] Colwell R K EstimateS: Statistical estimation of species richness and shared species from samples. 9.1.0.. https://www.robertkcolwell.org/pages/1407-estimates.

[B14237672] Duan Y, Zhu W, Chen S, Yan W, Zeng Z, Chen Y (2025). Infrared camera-based investigation of mammalian diversity in Hainan Tropical Rainforest National Park, China. National Park.

[B14237442] Fisher Jason T. (2023). Camera trapping in ecology: A new section for wildlife research. Ecology and Evolution.

[B14237684] Fu L, Bai W, Guo Z, Huang Y, LiYan W, Yang B, Hou J, Dong X, Zhang J, Zhou C (2020). Biodiversity and spatio-temporal patterns of mammals in the Mabian Dafengding National Nature Reserve using camera traps. Sichuan Journal of Zoology.

[B14264119] Gao X, Pan S, Sun Z, Li X, Gao T, Dong L, Wang N (2024). Distribution and activity rhythm of Small Indian Civet (*Viverricula
indica*) in Fenghuang Hill and Qi'ao Island, Zhuhai, Guangdong. Biodiversity Science.

[B14237699] Gui J, Cao Y, Pan D, Li J, Li K, Liu X, Li S, Yang M, Yang D (2025). Infrared camera monitoring and daily activity rhythm analysis of birds and mammals in the Hunan Dupangling National Nature Reserve. Acta Theriologica Sinica.

[B14264108] Han Q, Liang T, Zhang M, Liu M, Wang Z, Lu C (2022). Analysis of activity rhythm and habitat selection of Water Deer based on the infrared-triggered camera technology in autumn and winter in Urban Forest Park. Chinese Journal of Ecology.

[B14237810] He B, Sun R, Chen P, Dong W, Wang J, Wang D, Li S (2016). Baseline survey of mammal and bird diversity using camera-trapping in the Changqing National Nature Reserve of Shanxi Province. Acta Theriologica Sinica.

[B14237713] Hou D, Xu J, Li J, Jiang L, Chen Z, Duan Y, Wang R, Yu Z, Yu J (2022). Diurnal activity rhythm of sympatric Pheasants in Xianxialing Zhejiang Province. Journal of Northeast Forestry University.

[B14237764] Huang Y (2023). Population size and activity patterns of the Masked Palm Civet (*Paguma Larvata*) in the Fujian Junzifeng National Nature Reserve. Journal of Huaibei Normal University (Natural Sciences).

[B14237727] Hu J, Xie P, Li T, Guo R, Xu L, Song X, Xu A (2022). Temporal and spatial niche differentiation of sympatric Black Muntjac and Reeves' Muntjac. Acta Theriologica Sinica.

[B14237739] Hu J, Xie P, Mei Y, Guo R, Song X, Li T, Xu A (2023). Potential suitable habitats prediction and overlap analysis of Black Muntjac (*Muntiacus
crinifrons*) and Small Muntjac (*M.
reevesi*) in Qingliangfeng National Nature Reserve，Zhejiang Province. Acta Ecologica Sinica.

[B14238752] IUCN The IUCN Red List of Threatened Species.. https://www.iucnredlist.org/.

[B14237773] Ji Y, Yu Z, Yu J, Chen Z, Tong Z, Li D, Liu F (2022). Using camera traps to survey mammals and birds in Zhejiang Jiangshan Xianxialing Provincial Nature Reserve. Acta Theriologica Sinica.

[B14237451] John Christian, Post Eric (2021). Seasonality, niche management and vertical migration in landscapes of relief. Ecography.

[B14237798] Li A, Li Y, Shao R, Qian L, Shen J, Zhang C, Ta Q (2022). Analysis of activity patterns of the Chinese Ferret Badger using infrared camera technology. Chinese Journal of Zoology.

[B14237822] Li H, Li T, Deng Z, Chen R, Zhu W, Ren S, Wang Z (2023). Investigating bird and animal resources and rhythm characteristics in Wudaoxia Nature Reserve with infrared cameras. Forest Inventory and Planning.

[B14294692] Li J, Xu H, Wan Y, Sun J, Li S, Cai L (2018). Progress in construction of China mammal diversity observation network (China BON-Mammals). Journal of Ecology and Rural Environment.

[B14238691] Liu S, Wu Y, Li S (2022). Chinese mammal illustratedcatalogue.

[B14237858] Liu X, Wu P, He X, Zhao X (2018). Application and data mining of infrared camera in the monitoring of species. Biodiversity Science.

[B14264401] Lu P, Lei M, Zhang H, Wang X, Zhao X (2003). Study on sexual dimorphism of the Taihang Rhesus Macaque by discriminant function analysis. Shanghai Laboratory Animal Science.

[B14294411] Nie Y, Zhang C, Gao H, Liu H, Xie B (2025). Advances in conservation research of large- and medium-sized terrestrialmammals in China. Acta Theriologica Sinica.

[B14425387] Ricotta Carlo, Feoli Enrico (2024). Hill numbers everywhere. Does it make ecological sense?. Ecological Indicators.

[B14239646] Shannon C. E. (1948). A mathematical theory of communication. The Bell System Technical Journal.

[B14237893] Si H, Jin Z, Zhang K, Zhou F, Yao Y, Xiao H, Li B, Pucuowangjia, Xu H (2023). Seasonal differences in habitat selection of Rhesus Macaques (*Macaca
mulatta*) in the Western Sichuan Plateau Region. Acta Theriologica Sinica.

[B14239975] Simpson E. H. (1949). Measurement of diversity. Nature.

[B14237907] Song X, Tang W, Zhang Y (2017). Freshwater fish fauna and zoogeographical divisions in the Wuyi-Xian-xialing Mountains of Eastern China. Biodiversity Science.

[B14237929] Wang C, Li H, Yang Z, Bi X, Fan H, Su H, Hu C, Zhang M (2022). Temporal and spatial distribution patterns of *Rhinopithecus Brelichi* and *Macaca
thibetana* in the same region of the Northeast Fanjingshan. Scientia Silvae Sinicae.

[B14238845] Wang X, Chen J (2026). Camera trap survey of mammal diversity and activity rhythms of threatened species in Xianxialing Nature Reserve, Zhejiang Province, China. Chinese Academy of Sciences (CAS).Sampling event dataset.

[B14238699] Wei F, Yang Q, Wu Y, Jiang X, Liu S, Hu Y, Ge D, Li B, Yang G, Li M, Zhou J, Li S, Li S, Yu W, Chen B, Zhang Z, Zhou C, Wu S, Zhang L, Chen Z, Chen S, Deng H, Jiang T, Zhang L, Shi H, Lu X, Li Q, Liu Z, Cui Y, Li Y, He K (2025). Catalogue of mammals in China (2024). Acta Theriologica Sinica.

[B14238080] Wu P, Liu X, Cai Q, He X, Melissa S, Zhu Y, Shao X (2012). The application of infrared camera in mammal research in Guanyinshan Nature Reserve, Shanxi. Acta Theriologica Sinica.

[B14238169] Xiao C, Pan D, Zhu X, Huang Y, He Y, Xu J (2024). Analysis of the current habitat suitability of Chinese Serow in Anji County. Terrestrial Ecosystem and Conservation.

[B14239593] Xiao Z, Li X, Xiang Z, Li M, Jiang X, Zhang L (2017). Overview of the mammal diversity observation network of Sino BON. Biodiversity Science.

[B14238180] Yang C, Wan Y, Huang X, Yuan X, Zhou H, Fang H, Li D, Li J (2021). Activity rhythm of *Muntiacus
reevesi* based on infrared camera technology. Journal of Guangxi Normal University (Natural Science Edition).

[B14238193] Yu G, Li K, Chen R, Miao C, Wang G (2011). The study on habitat selection of Masked Palm Civet in autumn and winter in Hubei Wufeng Houhe National Nature Reserve. Chinese Journal of Wildlife.

[B14238203] Yu Z, Jin W, Chen Z, Tong Z, Liu B, Zhou X (2023). Investigation on mammals in Jiangshan Xianxialing Provincial Nature Reserve. Journal of Zhejiang Forestry Science and Technology.

[B14232142] Zeng G, Zheng H, Deng T (2009). Summer cave selection of Hog badger (*Arctonyx
collaris*) on the North Slopeof Funiu Mountain. Acta Ecologies Sinica.

[B14232165] Zeng G, Zheng H, Deng T (2010). A preliminary analysis on summer caves selection of Masked palm civet (*Paguma
larvata*) on the North Slope of Funiu Mountain. Acta Ecologica Sinica.

[B14238735] Zhang D (2022). Research on large mammal diversity and temporal-spatial pattern of the mammal activity using infrared camera-take in the Giant Panda National Park of Jiuzhaigo.

[B14238226] Zhang J, Xiong H, Liu X, Wu Y, Ma Y, Xia J, Hu X, Chen H, Chen W, Yang J (2025). The cluster behavior and activity rhythm of *Muntiacus
crinifrons* in Jiangxi Wuyuan Forest Bird National Nature Reserve. Journal of Anhui Agricultural Sciences.

[B14238241] Zhao J, Gong X, Pan J, Li Y, Wen Z, Ye Y, Gao J, Zhao A, Lin Z (2024). Comparative study on the spatial and temporal niche of the same do‐main distribution of *Arctonyx
collaris* and *Paguma
larvata* in Suichang, Zhejiang Province. Acta Theriologica Sinica.

[B14237473] Zhao Wei, Li Meng-Meng, Yan Xin-Rui, Zhang Zheng, Chen Xiao-Chun, Chang Zhao-Qian, Chen Jia-Long, Ma Jun-Jie, Xu Zhe-Ping, Chen Jin-Min, Lee Ping Shin, Zhang Fang (2026). Camera trap survey of mammal diversity and activity rhythms of threatened species in a subtropical forest of Huangshan Mountain, China. Biodiversity Data Journal.

