# Supplementary material for: Long-term camera trap monitoring of mammalian diversity and activity rhythms of threatened species in Xianxialing Nature Reserve, Zhejiang Province, China
Source: Biodivers Data J. 2026 Sep 8;14:e208779. doi: 10.3897/BDJ.14.e208779 (PMC13579192; doi:10.3897/BDJ.14.e208779)
Supplement: Supplementary file 1 [file bdj-14-e208779-s002.pdf]

Table S2. List of mammal species recorded at the Xianxialing Nature Reserve, Jiangshan of Zhejiang Province.

Species Name	Common Name	Geography	IUCN	CSRL	National Protected
**ARTIODACTYLA**					
**Bovidae**					
Capricornis milneedwardsii	Chinese Serow	O	VU	VU	II
**Cervidae**					
Elaphodus cephalophus	Tufted Deer	O	NT	NT	II
Muntiacus reevesi	Reeves' Muntjac	O	LC	NT	-
Muntiacus crinifrons	Black Muntjac	O	VU	EN	I
**Suidae**					
Sus scrofa	Wild Boar	Pa	LC	LC	-
**CARNIVORA**					
**Canidae**					
Canis lupus	Grey Wolf	Pa	LC	NT	II
Cuon alpinus	Dhole	Pa	EN	EN	I
Nyctereutes procyonoides	Racoon Dog	Pa	LC	NT	II
Vulpes vulpes	Red Fox	Pa	LC	NT	II
**Felidae**					
Neofelis nebulosa	Clouded Leopard	O	VU	CR	I
Prionailurus bengalensis	Leopard Cat	O	LC	VU	II
Pardofelis temminckii	Asiatic Golden Cat	O	NT	EN	I
Panthera pardus	Leopard	O	VU	EN	I
**Mustelidae**					
Arctonyx collaris	Hog Badger	O	LC	NT	-
Martes flavigula	Yellow-throated Marten	Pa	LC	VU	II
Mustela kathiah	Yellow-bellied Weasel	O	LC	NT	S
Melogale moschata	Chinese Ferret-badger	O	LC	NT	-
Mustela sibirica	Siberian Weasel	Pa	LC	LC	S
Meles leucurus	Asian Badger	Pa	NT	NT	-
**Herpestidae**					
Herpestes urva	Crab-eating Mongoose	O	LC	VU	S
**Ursidae**					
Ursus thibetanus	Asiatic Black Bear	O	VU	VU	II
**Viverridae**					
Paguma larvata	Masked Palm Civet	O	LC	NT	S
Viverricula indica	Small Indian Civet	O	NT	NT	I
**CHIROPTERA**					
**Hipposideridae**					
Hipposideros armiger	Greater Leaf-nosed Bat	O	LC	LC	-
Hipposideros pratti	Pratt's Roundleaf Bat	O	LC	NT	-
**Rhinolophidae**					

Rhinolophus luctus	Woolly Horseshoe Bat	O	LC	NT	-
Rhinolophus affinis	Intermediate Horseshoe Bat	O	LC	LC	-
Rhinolophus pearsonii	Pearson's Horseshoe Bat	O	LC	LC	-
Rhinolophus pusillus	Least Horseshoe Bat	O	LC	LC	-
Rhinolophus sinicus	Chinese Horseshoe Bat	O	LC	LC	-
**Vespertilionidae**					
Eptesicus serotinus	Oriental Serotine	O	LC	LC	-
Myotis chinensis	Chinese Myotis	O	LC	NT	-
Myotis laniger	Chinese Water Myotis	Pa	LC	LC	-
Miniopterus fuliginosus	Asian Long-fingered Bat	O	LC	NT	-
Nyctalus plancyi	Chinese Noctule	Pa	LC	LC	-
Pipistrelles abramus	Japanese Pipistrelle	O	LC	LC	-
Scotomanes ornatus	Emarginate Harlequin Bat	O	LC	LC	-
**EULIPOTYPHLA Erinaceidae**					
Erinaceus amurensis	Amur Hedgehog	Pa	LC	LC	-
**Soricidae**					
Crocidura attenuata	Asian Gray Shrew	O	LC	LC	-
Crocidura shantungensis	Asian Lesser White-toothed Shrew	Pa	LC	LC	-
Suncus murinus	Musk Shrew	O	LC	LC	-
**Talpidae**					
Mogera latouchei	La Touche's Mole	O	LC	LC	-
**LAGOMORPHA Leporidae**					
Lepus sinensis	Chinese Hare	O	LC	LC	-
**PRIMATES Cercopithecidae**					
Macaca mulatta	Rhesus Macaque	O	LC	LC	II
Macaca thibetana	Tibetan Macaque	O	NT	VU	II
**PHOLIDOTA Manidae**					
Manis pentadactyla	Chinese Pangolin	O	CR	CR	I
**RODENTIA Cricetidae**					

Eothenomys melanogaster	David's Chinese Vole	O	LC	LC	-
**Hystricidae**					
Hystrix hodgsoni	Malayan Porcupine	O	LC	LC	-
**Muridae**					
Apodemus agrarius	Striped Field Mouse	O	LC	LC	-
Apodemus draco	South China Field Mouse	O	LC	LC	-
Berylmys bowersi	Bower's White-toothed Rat	O	LC	LC	-
Leopoldamys edwardsi	Edward's Leopoldamys	O	LC	LC	-
Micromys minutus	Eurasian Harvest Mouse	O	LC	LC	-
Mus musculus	House Mouse	O	LC	LC	-
Niviventer fulvescens	Chestnut White-bellied Rat	O	LC	LC	-
Niviventer confucianus	Confucian Niviventer	O	LC	LC	-
Rattus tanezunmi	Oriental House Rat	O	LC	LC	-
Rattus norvegicus	Brown Rat	O	LC	LC	-
Rattus nitidus	White-footed Indochinese Rat	O	LC	LC	-
**Sciuridae**					
Callosciurus erythraeus	Pallas's Squirrel	O	LC	LC	-
Dremomys pernyi	Perny's Long-nosed Squirrel	O	LC	LC	-
Tamiops maritimus	Maritime Striped Squirrel	O	LC	LC	-
**Spalacidae**					
Rhizomys sinensis	Chinese Bamboo Rat	O	LC	LC	-

Note: O: Oriental realm, Pa: Palearctic realm; CR: Critically Endangered, EN: Endangered, VU: Vulnerable, NT: Near Threatened, LC: Least Concern; I: National First-Class Key Protected Wild Animals, II: National Second-Class Key Protected Wild Animals, S: Zhejiang Provincial Key Protected Wildlife. Source: The Comprehensive Scientific Expedition Report on Zhejiang Xianxialing Nature Reserve, 2021
